# Biosensors for the Detection of Interaction between *Legionella pneumophila* Collagen-Like Protein and Glycosaminoglycans

**DOI:** 10.3390/s18082668

**Published:** 2018-08-14

**Authors:** Han Su, Shaopei Li, Mauricio Terebiznik, Cyril Guyard, Kagan Kerman

**Affiliations:** 1 Department of Physical and Environmental Sciences, University of Toronto Scarborough, 1265 Military Trail, Toronto, ON M1C 1A4, Canada; hanson.su@mail.utoronto.ca (H.S.); shaopei.li@mail.utoronto.ca (S.L.); 2 Department of Biological Sciences and Cell and Systems Biology, University of Toronto Scarborough, 1265 Military Trail, Toronto, ON M1C 1A4, Canada; terebiznik@utsc.utoronto.ca; 3 BIOASTER Microbiology Technology Institute, 40 Avenue Tony Garnier, 69007 Lyon, France; cyril.guyard@bioaster.org

**Keywords:** Legionella collagen-like protein, glycosaminoglycans, fucoidan, surface plasmon resonance, electrochemical impedance spectroscopy

## Abstract

The adhesin Legionella collagen-like (Lcl) protein can bind to extracellular matrix components and mediate the binding of *Legionella pneumophila* to host cells. In this study, electrochemical impedance spectroscopy (EIS) and surface plasmon resonance (SPR)-based biosensors were employed to characterize these interactions between glycosaminoglycans (GAGs) and the adhesin Lcl protein. Fucoidan displayed a high affinity (K_D_ 18 nM) for Lcl protein. Chondroitin sulfate A and dermatan sulfate differ in the position of a carboxyl group replacing D-glucuronate with D-iduronate. Our results indicated that the presence of D-iduronate in dermatan sulfate strongly hindered its interaction with Lcl. These biophysical studies provided valuable information in our understanding of adhesin-ligand interactions related to *Legionella pneumophila* infections.

## 1. Introduction

*Legionella pneumophila* is a Gram-negative, facultative intracellular pathogen and the causative agent of Legionnaire’s disease. The latter could lead to severe pneumonia and even death, if not treated at an early stage [1,2,3]. Untreated immunosuppressed patients have a 40% to 60% chance of fatality [4]. *L. pneumophila* is ubiquitously found in natural and human-made fresh water reservoirs and distribution systems. The natural hosts of *L. pneumophila* are amoebae and replication occurs within the hosts after phagocytosis. *L. pneumophila* infections occur when contaminated airborne water droplets are inhaled into the lung allowing the bacteria to reach the alveolar mucosa [3,5]. Recently, the molecular basis of biofilm formation by *L. pneumophila* was reported along with the role of other microbial species in *L. pneumophila* biofilm colonization [6]. Bacterial aggregates of *L. pneumophila* have been reported to have the ability to resist various host defenses and colonize their biofilm environment efficiently [7]. Recent studies have hypothesized that *Legionella* collagen-like (Lcl) protein induced the auto-aggregation process in a divalent-cation-dependent manner. The isolates from *Legionella* species which did not produce Lcl, were deficient in auto-aggregation [7].

The extracellular matrix of alveolar mucosa contains multiple proteinaceous and non-protein components that are thought to play critical roles in the etiology and pathogenesis of Legionnaire’s disease. One of these components, the sulfated glycosaminoglycans (GAGs), includes heparan sulfate, chondroitin sulfate, and keratan sulfate. The sulfated GAGs are negatively-charged heteropolysaccharides expressed in all mammalian tissues. Enzymatically-generated structural patterns and the degree of sulfation in GAGs determine their specific interactions with protein ligands. Chondroitin sulfate and fucose were reported to bind to Lcl using enzyme-linked immunoassays (ELISAs) [8,9]. In order to shed light on the interaction of GAGs with Lcl protein, we have assessed the adhesin-ligand interactions from a perspective of electrochemical impedance spectroscopy (EIS) and surface plasmon resonance (SPR). SPR is a widely used analytical tool for studying interactions between ligands and analytes. The sensitivity and simplicity of SPR provide many advantages in applications such as drug screening as well as biomolecular interaction studies [10,11]. In this report, Lcl proteins were immobilized as ligands onto the gold sensor chip surface, where they could interact with the incoming GAGs as adhesins. The real-time data were collected and formulated into the kinetic information of the affinity (*K*_D_) between Lcl and GAGs. Similarly, EIS is also a well-established electro-analytical method to investigate biomolecular interactions. Previous studies have employed EIS in studying protein interactions in neurodegenerative diseases, as well as biological analysis [12,13,14]. As for this EIS-based study, Lcl proteins were immobilized onto a gold screen-printed electrode surface, where the binding of GAGs was determined by following the changes in the charge-transfer resistance values of the Randles’ equivalent circuit [12,13,14,15,16,17]. Together, both SPR and EIS offered a cost-effective and rapid approach to study protein-GAG interactions with high sensitivity and low sample consumption.

## 2. Materials and Methods

Recombinant, N-terminus His-tagged Lcl proteins were prepared following the procedures as reported by C. Guyard and co-workers [6,7]. Designs and constructs of Lcl proteins were kindly donated by C. Guyard (BIOASTER, Microbiology Technology Institute, Lyon, France). HEPES, sodium chloride, EDTA, nickel (II) chloride, 3,3′-dithiobis (sulfosuccinimidyl) propionate, *N*-(5-amino-1-carboxypentyl) iminodiacetic acid (ANTA), sodium phosphate monobasic (NaH_2_PO_4_; 99%), sodium phosphate dibasic (Na_2_HPO_4_; 99.0%), and ethanol-amine were purchased from Sigma-Aldrich (Oakville, ON, Canada). All other reagents and chemicals were of analytical grade.

### 2.1. Surface Plasmon Resonance (SPR)

SPR-based analyses were performed using a Biacore X100 (GE Healthcare, Chicago, IL, USA) with a Series S NTA sensor chip on a dextran support. All measurements were conducted at 25 °C and all the solutions were sterile filtered (0.2 μm) before injection into the flow cells. SPR running buffer was composed of 0.01 M HEPES (pH 7.4), 0.15 M NaCl, 0.05 mM EDTA with 0.05% surfactant *P*20. The nickel solution contained 0.5 mM NiCl_2_ in running buffer. The regeneration solution was composed of 0.01 M HEPES (pH 8.3), 0.15 M NaCl, 0.35 M EDTA, and 0.05% surfactant *P*20.

**Scheme 1:**
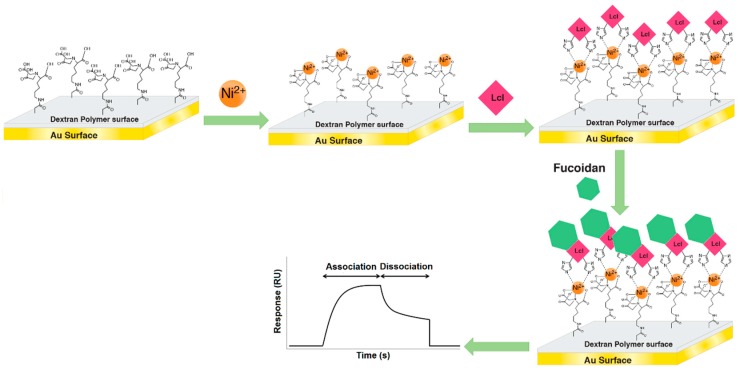
Illustrative representation of Lcl immobilization and fucoidan detection using surface plasmon resonance (SPR).

Two flow cells of the sensor chip were used, one (reference flow cell, FC-1) to detect non-specific binding and background subtraction for the other one (detection flow cell, FC-2), which had the immobilized Lcl protein. The system was washed extensively with regeneration buffer for 180 s at a flow rate of 10 μL/min, followed by a wash with running buffer at 30 μL/min until the baseline became stable. As shown in Scheme 1, the solution of Ni^2+^ was injected into both flow cells at 10 μL/min for 60 s to saturate the NTA surface with Ni^2+^. His-tagged Lcl protein in running buffer (1 μM) was exposed to FC-2 at 10 μL/min for 1080 s. The GAGs were injected into both flow cells at 1 μM, at a flow rate of 30 μL/min for 120 s, for each cycle. The setup was automated using the Biacore X100 Evaluation software (GE Healthcare, Chicago, IL, USA). All interaction measurements as well as the control measurements with blank buffer solution before and after each injection of analytes were performed in triplicate.

### 2.2. Electrochemical Impedance Spectroscopy (EIS)

**Scheme 2:**
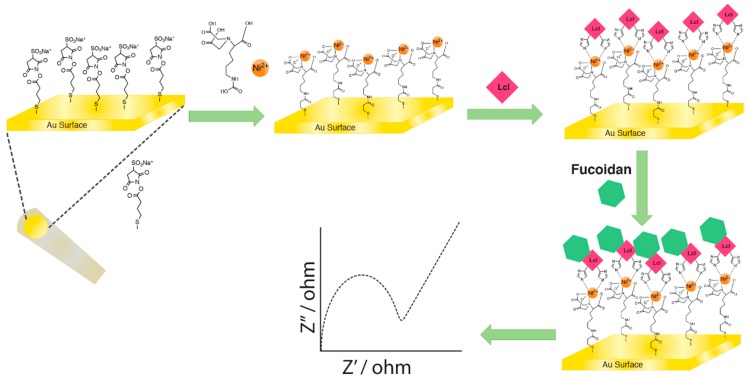
Illustrative representation of Lcl immobilization and fucoidan detection using electrochemical impedance spectroscopy (EIS).

A gold compact-disc electrode was constructed and used as the working electrode in EIS measurements as reported by Veloso et al. [18]. The electrodes were prepared using Kodak™ (Rochester, NY, USA) gold preservation archival grade CDs, consisting of a 50–100 nm gold film on a photosensitive layer on top of a polycarbonate disc. The protective polymer, coating the gold surface, was removed with the application of 5 mL of concentrated nitric acid on the CD surface for 10 min. The polymer and excess nitric acid was rinsed with distilled water. The CD gold surface was cut into rectangular chips with dimensions of 1 cm width × 2 cm length. An insulating tape with a hole of 2 mm^2^ area was glued on the gold surface to isolate the working area [18,19]. As shown in Scheme 2, the immobilization of His-tagged proteins onto the gold CD chips was accomplished by the Ni^2+^/NTA complex, which was linked to the gold surface using a homobifunctional linker, 3,3′-dithiobis (sulfosuccinimidyl) propionate (DTSSP) [20].

Immobilization of the DTSSP layer was accomplished by depositing an aliquot (20 μL) of 2 mM DTSSP in 100 mM Na_2_CO_3_ (pH 8.5) onto the gold surface overnight at 4 °C, followed by a rinse with Na_2_CO_3_ to remove excess DTSSP [20,21]. The formation of Ni^2+^/NTA complex (5 μM NiCl_2_, 2 μM NTA in 100 mM Na_2_CO_3_, pH 8.5, incubated for 4 h) was achieved by depositing an aliquot (20 μL) of the Ni^2+^/NTA solution on the electrode surface for overnight incubation at room temperature 25 °C. After rinsing off the excess Ni^2+^/NTA solution with Na_2_CO_3_, 0.1 M ethanolamine was used to block the unreacted N-hydroxy (sulfo)succinimide active esters for 1 h at room temperature, which was then rinsed off with 50 mM phosphate-buffered saline (PBS, pH 7.4) with 100 mM NaCl [17]. An aliquot (20 µL) of the His-tagged Lcl protein (1 μM in 0.01 M HEPES (pH 7.4), 0.15 M NaCl, 0.05 mM EDTA with 0.05% surfactant *P*20) was then added to the electrode surface and incubated overnight at room temperature, followed by rinsing with HEPES buffer (pH 7.4). This sensor chip will be referred to as “control”, on which analytes were further exposed to test for non-specific binding. GAGs at 1 μM were then deposited onto the surface and incubated overnight at room temperature, which was then rinsed with HEPES buffer (pH 7.4). Triplicate sensors of each GAGs and controls were prepared to obtain the mean data and uncertainties of the measurements. Electrochemical measurements were conducted using a µAutolab-III potentiostat (Metrohm Autolab BV, Utrecht, The Netherlands) in a three-electrode configuration consisted of a gold CD working electrode, Ag/AgCl reference electrode (in 3 M KCl) and a platinum counter electrode. EIS measurements were performed using the Frequency Response Analysis (FRA) system software, over a frequency range of 0.1 to 100,000 Hz with 0.005 V amplitude (rms). A Randles equivalent circuit model was employed to fit the impedance data using FRA in order to determine the changes in charge transfer resistance (R_CT_) due to the modifications on the electrode surface. An increase in R_CT_ generally results from the binding of biomolecules to the surface due to the increase in resistance for transfer of electrons from the solution to the electrode surface.

## 3. Results and Discussion

**Figure 1:**
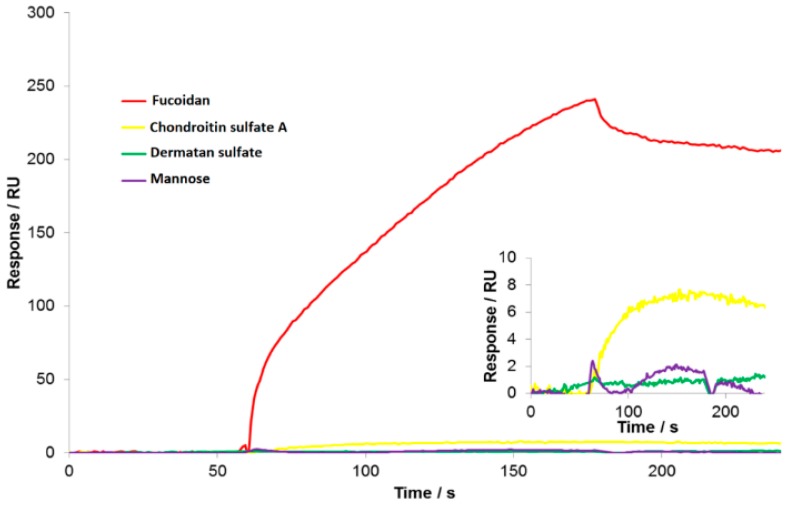
SPR sensorgrams displaying the binding of the glycosaminoglycans (GAGs), at 1 μM with His-tagged Lcl proteins immobilized on the NTA-modified gold sensor chip surface. Other conditions were as described in the Experimental section.

His-tagged Lcl proteins were attached to the Ni-NTA surface (Supplementary Figure S2) on the sensor chips to allow further interactions with the analytes. As the GAGs were exposed to the surface-immobilized Lcl protein, the SPR angle shifted, which, in turn, increased the response unit. When the flow of GAGs was stopped, blank buffer solution would be exposed to the chip surface to wash away the unbound and non-specifically attached molecules. The resulting response would be the net increase due to the specific interaction of the GAGs with Lcl protein. The sensorgram in Figure 1 shows the real-time response data, which was used to calculate the value of *K*_D_ using the Biacore Evaluation Software™. Fucoidan, a close analog to fucose, was observed to give a significantly large signal increase relative to the other GAGs. This is a result of the strong interaction between fucoidan and Lcl, and the relatively larger in size of fucoidan (50 kDa) in contrast to the rest of the GAGs used in this study. Both mannose and dermatan sulfate showed significantly small signal response, which fell in the noise range, and the shape of SPR response was indicative of no or negligible interaction with the surface-bound Lcl proteins.

**Figure 2:**
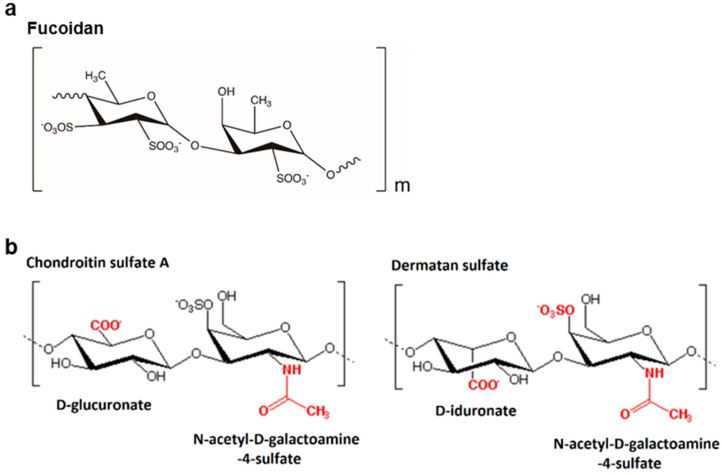
(**a**) Chemical structure of fucoidan and (**b**) chemical structures of chondroitin sulfate A (chondroitin-4-sulfate) and dermatan sulfate highlighting the position of functional groups in red.

The binding affinity (K_D_) was calculated from SPR data for the binding of fucoidan, chondroitin sulfate A, dermatan sulfate, and mannose to the Lcl protein immobilized on the sensor chip. The values with dermatan sulfate and mannose were so large that it exceeded the limits of the software, indicating a very low affinity interaction, and thus, labeled as “not detectable”. Mannose was used as a negative control, as it was not expected to have any affinity towards Lcl protein due to the lack of negatively charged sulfate groups. Fucoidan, as shown on Figure 2a, a close analog to fucose, which is found commonly in human lung epithelial cells, was tested as a positive control. Fucoidan measurements displayed a very strong affinity with a K_D_ value of 18 ± 2.1 nM. Chondroitin sulfate A also displayed a strong affinity with a K_D_ value of 173 ± 24 nM. Although chondroitin sulfate A and dermatan sulfate both have the negatively charged carboxyl groups, they differ in the position of carboxyl group replacing D-glucuronate with D-iduronate as shown in Figure 2b. We hypothesized that the presence of D-iduronate in dermatan sulfate hindered its interaction with Lcl. Furthermore, in order to support specificity of such interactions, fucoidan and chondroitin sulfate A were injected onto a non-Lcl immobilized, Ni^2+^-activated NTA sensor chip. The resulting response is shown in Supplementary Figure S1 to demonstrate the non-specific interactions of the analytes on the sensor chip.

**Figure 3:**
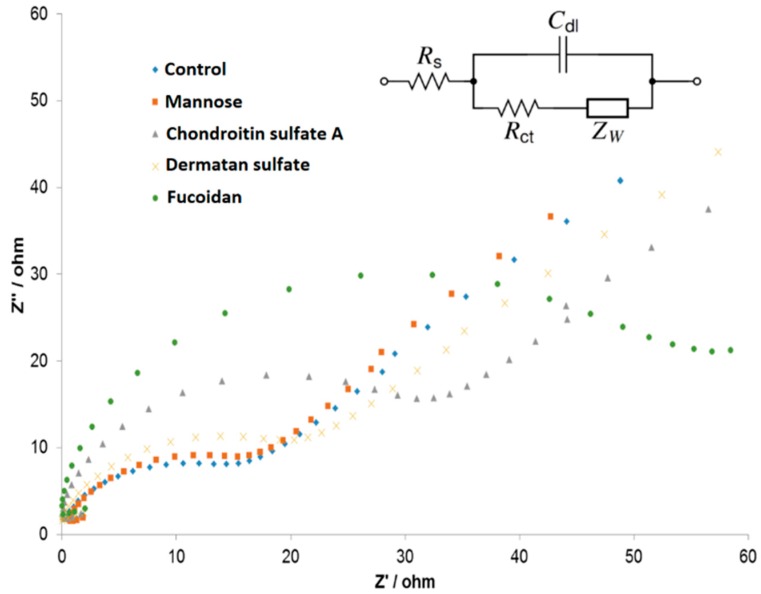
Representative Nyquist plots displaying the interaction of GAGs at 1 µM with His-tagged Lcl proteins that were covalently immobilized on gold CD chips. The inset shows the Randles equivalent circuit. Other conditions are as described in the Experimental section.

For EIS measurements, the incubation of GAGs with the Lcl-modified gold electrode surfaces allowed the attachment of GAGs to the protein. Followed by a wash with buffer solution, the non-specifically bound molecules were removed from the surface to avoid false positive signals for the EIS measurements. As shown in Figure 3, both mannose and dermatan sulfate showed negligible EIS signals similar to those of the blank samples (no analyte), indicating that these molecules did not have a significant affinity to the surface-bound Lcl proteins and were easily rinsed away by the buffer. Fucoidan and chondroitin sulfate A showed a significant increase in the EIS signals after thorough rinsing, demonstrating a strong binding that occurred between Lcl proteins and GAGs on the electrode surface.

The EIS data fitting parameters in Table 1 demonstrated that our results were in agreement with the ones observed using SPR, with fucoidan showing the highest charge transfer resistance, indicating the strong attachment of the molecules to the electrode surface. The unbound analytes were rinsed off from the surface and showed negligibly low changes in charge transfer resistance (15.62 Ω and 16.10 Ω, respectively) in comparison with the blank sample measurements.

Both the SPR and EIS data were in agreement with the previously reported ELISA-based results by Guyard and co-workers [8,9] in terms of relative affinities between Lcl proteins with GAGs. Currently, our group is studying various parameters for discovering the exact pathway and mechanism in which *L. pneumophila* can attach to the host cells. Biomolecules, including heparan sulfate, collagen, and fibronectin, are also under investigation using the described SPR and EIS techniques in this report. These biomolecules are also common components of the extracellular matrix, which can play an important role in the interaction of Lcl proteins with the host cells during an infection.

## 4. Conclusions

To the best of our knowledge, this is the first attempt for the quantitative measurement of a binding interaction between GAGs and the Lcl protein using biosensors. In this proof-of-concept study, we have determined the strong affinity (*K*_D_) between Lcl and fucoidan. We have also observed that the position of the carboxyl group replacing D-glucuronate with D-iduronate in dermatan sulfate significantly prevents its interaction with Lcl. It has been suggested that a tandem repeat domain of the Lcl protein could be responsible for these affinity interactions and auto-aggregation [8,9]. The reported biosensors here can be easily adapted to screening and discovery of biomolecular therapeutics that can manipulate the interaction of *L. pneumophila* with lung cells during an infection.

## Figures and Tables

**Table 1:** Summary for the simulation of EIS fitting process according to the Randles equivalent circuit, in which the binding of GAGs with the surface-bound Lcl proteins was monitored on a gold CD chip surface. Blank samples contained 0.01 M HEPES buffer solution (pH 7.4), including 0.15 M NaCl, 0.05 mM EDTA, and 0.05% surfactant *P*20 in the absence of GAGs.

	R_CT_ (Ohm)	R_s_ (Ohm)	C_dl_ (μF)
Control	15.06 ± 1.82	23.62 ± 2.54	3.22 ± 0.35
Mannose	15.62 ± 1.74	27.40 ± 3.01	4.34 ± 0.49
Dermatan sulfate	16.10 ± 2.03	22.50 ± 2.29	11.14 ± 1.33
Chondroitin sulfate A	45.90 ± 4.96	29.43 ± 3.24	17.09 ± 1.98
Fucoidan	74.06 ± 8.11	25.53 ± 2.98	21.90 ± 2.43

## Supplementary Materials

The following are available online at http://www.mdpi.com/1424-8220/18/8/2668/s1.
