# Supplementary material for: Biosensors for the Detection of Interaction between Legionella pneumophila Collagen-Like Protein and Glycosaminoglycans
Source: Sensors (Basel). 2018 Aug 14;18(8):2668. doi: 10.3390/s18082668 (PMC6111780; doi:10.3390/s18082668)
Supplement: Supplementary file 1 [file sensors-18-02668-s001.pdf]

Biosensors for the detection of interaction between *Legionella pneumophila* collagen-like protein and glycosaminoglycans

Han Su [a], Shaopei Li [a], Mauricio Terebiznik [b], Cyril Guyard [c] and Kagan Kerman [a,*]

[a] Department of Physical and Environmental Sciences, University of Toronto Scarborough, 1265 Military Trail, Toronto, M1C 1A4, ON, Canada.

[b] Department of Biological Sciences and Cell and Systems Biology, University of Toronto Scarborough, 1265 Military Trail, Toronto, M1C 1A4, ON, Canada. terebiznik@utsc.utoronto.ca

[c] BIOASTER Microbiology Technology Institute, 40 Avenue Tony Garnier, 69007 Lyon, France. cyril.guyard@bioaster.org

[*] Corresponding author: kagan.kerman@utoronto.ca; Tel.: +1 416 287 7249

Supplementary Materials:

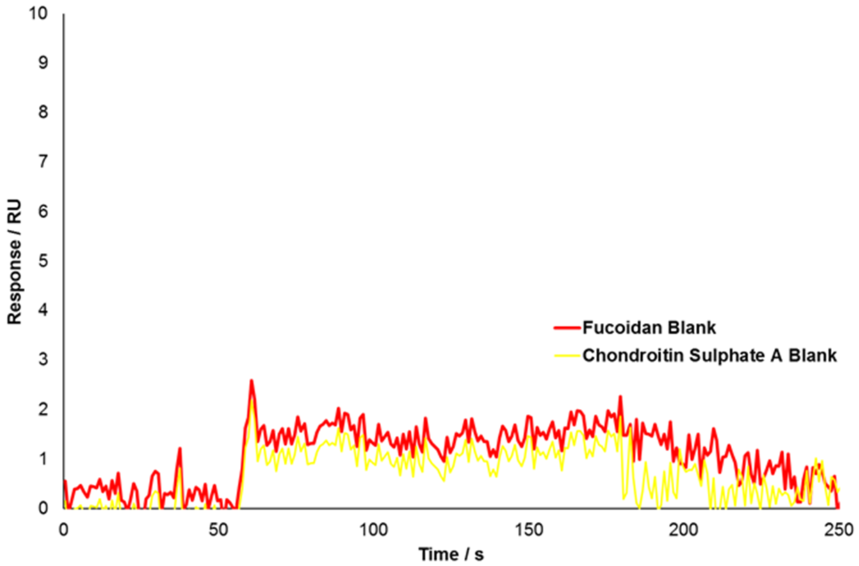

Figure S1: SPR responses of fucoidan and chondroitin Sulphate A on non-immobilized, Ni^{2+} activated NTA sensorchip. This data demonstrate that the non-specific interaction of the analytes with the Ni^{2+} activated NTA immobilized surface is negligible in the absence of His-rLcl.

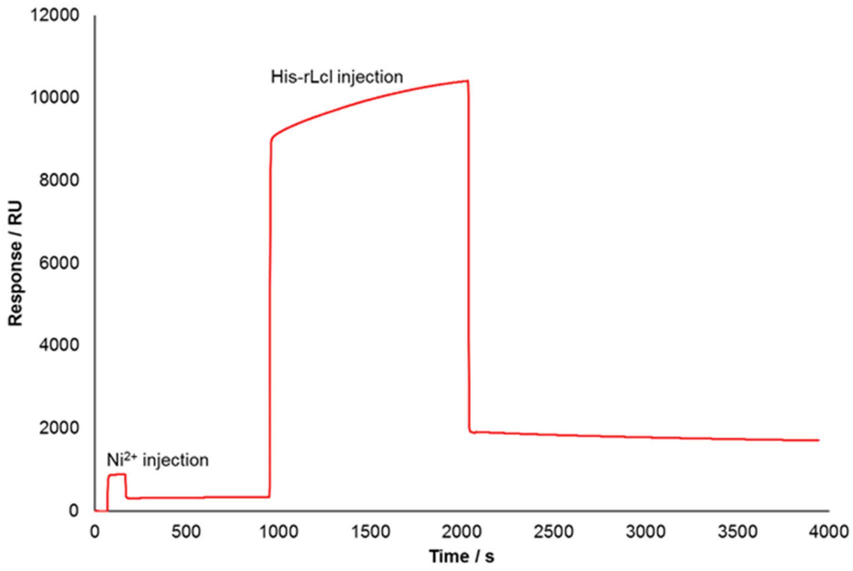

Figure S2: SPR responses of Ni^{2+} activation, followed by immobilization of His-rLcl. HEPES buffer solution was injected after each injection to remove non-specifically adsorbed biomolecules.
